# The Prognostic Value of Forkhead Box P3 Expression in Operable Breast Cancer: A Large-Scale Meta-Analysis

**DOI:** 10.1371/journal.pone.0136374

**Published:** 2015-08-25

**Authors:** Shu Chen Lin, Zhi Hua Gan, Yang Yao, Da Liu Min

**Affiliations:** 1 Department of Oncology, Shanghai Sixth People’s Hospital East Campus, Shanghai Jiao Tong University, Shanghai, People’s Republic of China; 2 Department of Oncology, The Sixth People’s Hospital, Shanghai Jiao Tong University, Shanghai, People’s Republic of China; National Cancer Center, JAPAN

## Abstract

**Background:**

Recent studies have shown that the forkhead box P3 (FOXP3) protein has a prognostic role in breast cancer. However, these results are controversial. Therefore, the aim of this meta-analysis was to clarify the prognostic role of FOXP3 expression in operable breast cancer cases.

**Methods:**

Eligible studies describing the use of FOXP3 as a prognostic factor for operable breast cancer cases were identified. Clinicopathological features, disease-free survival (DFS), and overall survival (OS) data were collected from these studies and were analyzed using Stata software.

**Results:**

A total of 16 articles containing data from 13,217 breast cancer patients met the inclusion criteria established for this study. The subsequent meta-analysis that was performed showed that high levels of FOXP3 are not significantly associated with DFS and OS with significant heterogeneity. An additional subgroup analysis demonstrated that intratumoral FOXP3+ regulatory T cells (Tregs) were positively correlated with adverse clinicopathological parameters, yet they did not show an association with DFS or OS. For tumor cells, the pooled results revealed that FOXP3 is significantly associated with DFS (HR: 2.55, 95% CI: 1.23–5.30) but is not associated with clinicopathological parameters or OS. We also observed a significant correlation between FOXP3 expression and survival in the estrogen receptor-positive (ER)+ subgroup (HR: 1.83, 95% CI: 1.36–2.47 for DFS, HR: 1.87, 95% CI 1.28–2.73 for OS), in the Asian region (HR: 1.98, 95% CI: 1.56–2.50 for DFS, HR: 1.93, 95% CI: 1.12–3.35 for OS) and using the median as the FOXP3-positive cut-off value (HR: 1.94, 95% CI: 1.57–2.39 for DFS, HR: 2.06; 95% CI: 1.36–3.11 for OS).

**Conclusion:**

This meta-analysis indicates that a prognostic role for FOXP3 expression in operable breast cancer cases depends on the FOXP3-positive region, ER status, geographic region and the FOXP3-positive cut-off value.

## Introduction

Forkhead box P3 (FOXP3) is a transcription factor with a highly conserved forkhead DNA-binding domain. CD4+/CD25+ regulatory T cells (Tregs) express FOXP3, and they exhibit a suppressor activity similar to that of many other immune cells, such as cytotoxic T-lymphocytes (CTLs), natural killer (NK) cells, NK T cells, B cells, macrophages, and dendritic cells [1]. Furthermore, strong evidence indicates that the tumor stroma may influence the malignant capacity of tumor epithelial cells and is thus actively involved in tumorigenesis [2]. Therefore, the infiltration of FOXP3 Tregs into tumor stroma may represent a critical factor for cancer immunity and could affect cancer progression. However, the data supporting these hypotheses have discrepancies [3].

The results reported in recent studies suggest that FOXP3 is not only expressed by lymphocytes, but is also expressed by normal epithelial cells and tumor cells [4]. The role of FOXP3 in tumor cells has been studied for many years. In vitro, FOXP3 represses the transcription of the *HER2*, *SKP2*, *MYC*, *MMP2*, and *uPA* genes and induces the expression of *p21* and *LATS2* [4]. Thus, inhibited cell growth, cell migration, and cell invasion have been observed in cell lines derived from breast, prostate, and ovarian cancers that overexpress FOXP3 [4]. Furthermore, in experimental animal models, the loss of FOXP3 expression in mammary and prostatic epithelial tissues leads to tumor formation [5]. Therefore, FOXP3 expression in tumor cells has been hypothesized to represent a favorable prognostic factor in human cancers. However, the results reported to date have been inconsistent [1].

To clarify the prognostic role of FOXP3 expression in breast cancer (BC), a meta-analysis was performed to systematically review papers published over the past decade that describe FOXP3 expression in relation to clinicopathological features and patient survival in BC cases [6–21].

## Materials and Methods

### Literature search

A systematic literature search of PubMed, EMBASE, and the Cochrane Library was performed to analyze the prognostic value of FOXP3 in BC patients. Relevant articles presented at the annual meetings of the European Society of Medical Oncology (ESMO) and the American Society of Medical Oncology (ASCO) were also reviewed. The search strategies employed subject headings, key words, and freedom words, and the list of publications was restricted to those published in English. The search terms included the following words, variously combined: “breast”, “mammary”, “cancer”, “tumor”, “tumour”, “carcinoma”, “neoplasm”, “adenocarcinoma”, “sarcoma”, “dcis”, “ductal”, “forkhead box P3,” “FOXP3”, “SCURFIN”, “IPEX”, “prognosis”, “outcome”, “progress”, “metastasis”, “relapse”, “survival” and et al.. The detailed search strategies are available in the S1 File. The final search was performed on December 25, 2014.

### Study selection, inclusion and exclusion criteria

Both reviewers (ShuChen Lin and ZhiHua Gan) initially checked the titles and abstracts of the publications to assess relevance. The full texts of the remaining studies were further assessed according to the following inclusion criteria: (1) a pathological diagnosis of operable BC was made, (2) an association between FOXP3 and overall survival (OS), disease-free survival (DFS), or clinicopathological features was described, and (3) the studies represented original articles. Reviews, comments, and book chapters were excluded. Cases of metastatic or local advanced disease with preoperative chemotherapy were also excluded. Duplicate studies were excluded by verifying the names of the authors and the study details. We contacted the authors for further information when needed. Any discrepancies were resolved by consensus.

### Quality assessment

To control the quality of the meta-analysis, two reviewers (ShuChen Lin and ZhiHua Gan) assessed the quality of each study using the Newcastle—Ottawa Quality Assessment Scale (NOS; Table 1) [22]. This scale provides scores according to patient population and selection, study comparability, follow-up, and outcome of interest. NOS scores of 1–3, 4–6 and 7–9 were defined as low-, intermediate- and high-quality studies, respectively. Any discrepancies were resolved by consensus.

**Table 1:** Newcastle—Ottawa quality assessment scale.

**Selection**
**(1) Representativeness of the exposed cohort**
**(a) Truly representative of the average ‘BC patient’ in the community (1 star)**
**(b) Somewhat representative of the average ‘BC patient’ in the community (1 star)**
**(c) Selected group of users (e.g. nurses, volunteers)**
**(d) No description of the derivation of the cohort**
**(2) Selection of the non-exposed cohort**
**(a) Drawn from the same community as the exposed cohort (1 star)**
**(b) Drawn from a different source**
**(c) No description of the derivation of the non-exposed cohort**
**(3) Ascertainment of exposure (Proof of BC and FOXP3 measurement)**
**(a) Secure record (eg surgical records) (1 star)**
**(b) Structured interview (1 star)**
**(c) Written self-report**
**(d) No description**
**(4) Demonstration that outcome of interest was not present at start of study**
**(a) Yes (1 star)**
**(b) No**
**Comparability**
**(1) Comparability of cohorts on the basis of the design or analysis**
**(a) Study controls for ‘metastasis or micrometastasis’ (1 star)**
**(b) Study controls for any additional factor (1 star) (Age, stage, grade etc.)**
**Outcome**
**(1) Assessment of outcome (Death or recurrence)**
**(a) Independent blind assessment (1 star)**
**(b) Record linkage (1 star)**
**(c) Self-report**
**(d) No description**
**(2) Was follow-up long enough for outcomes to occur? (Death or recurrence)**
**(a) Yes (‘1 years’) (1 star)**
**(b) No**
**(3) Adequacy of follow-up of cohorts**
**(a) Complete follow-up—all subjects accounted for (1 star)**
**(b) Subjects lost to follow-up unlikely to introduce bias—small number lost ‘(25%)’ or description provided of those lost (1 star)**
**(c) Follow-up rate ‘o75%’ and no description of those lost**
**(d) No statement**

BC: breast cancer.

### Data extraction

Data extraction was conducted independently by two reviewers (ShuChen Lin and ZhiHua Gan). The following information was collected: the first author’s last name, the publication year, the country in which the study was conducted, the tumor type, the sample size, the positive region, the cut-off value to assess FOXP3 positivity, the clinicopathological features, the survival data (including DFS and OS), the analysis method, and the study design. For incomplete data, follow-up information was estimated based on the accrual period, the median follow-up period, the date of analysis, and the date of submission, as described by Tierney et al. [23]. Any discrepancies were discussed and resolved by consensus.

### Statistical analysis

Pooled estimates of odds ratios (OR) were used to estimate the correlations between FOXP3 expression and the clinical parameters of BC, which included tumor size, lymph node metastasis, estrogen receptor (ER) positivity, Her-2 positivity, and histological grade. With respect to histology, good differentiation (G1) and moderate differentiation (G2) were combined, while poor differentiation (G3) constituted a second group. Hazard ratio (HR) information was extracted to estimate the association between FOXP3 and OS or DFS for BC patients. Engauge Digitizer version 4.1 software was used to extract survival data from Kaplan-Meier curves. Estimates of HR values were obtained according to previously described methods [23]. P-values and I^2^ values reflect data heterogeneity. Depending on these results, a fixed or random model was applied. A sensitivity analysis was used to investigate the influence of individual studies on the pooled HR by omitting one study at a time and recalculating the pooled HR. Subgroup stratification analyses were performed to identify sources of heterogeneity according to the FOXP3-positive region, the ER status, the sample size, the study design, the geographic region and the FOXP3-positive cut-off value. Publication bias was also investigated using Egger’s and Begg’s graphical methods [24, 25]. The nonparametric “trim and fill” approach was used to further assess the possible effect of publication bias in our meta-analysis [26]. All data analyses were performed using Stata version 12.0 software (Stata Corporation, College Station, TX, USA).

## Results

### Identification of eligible studies

**Fig 1:**
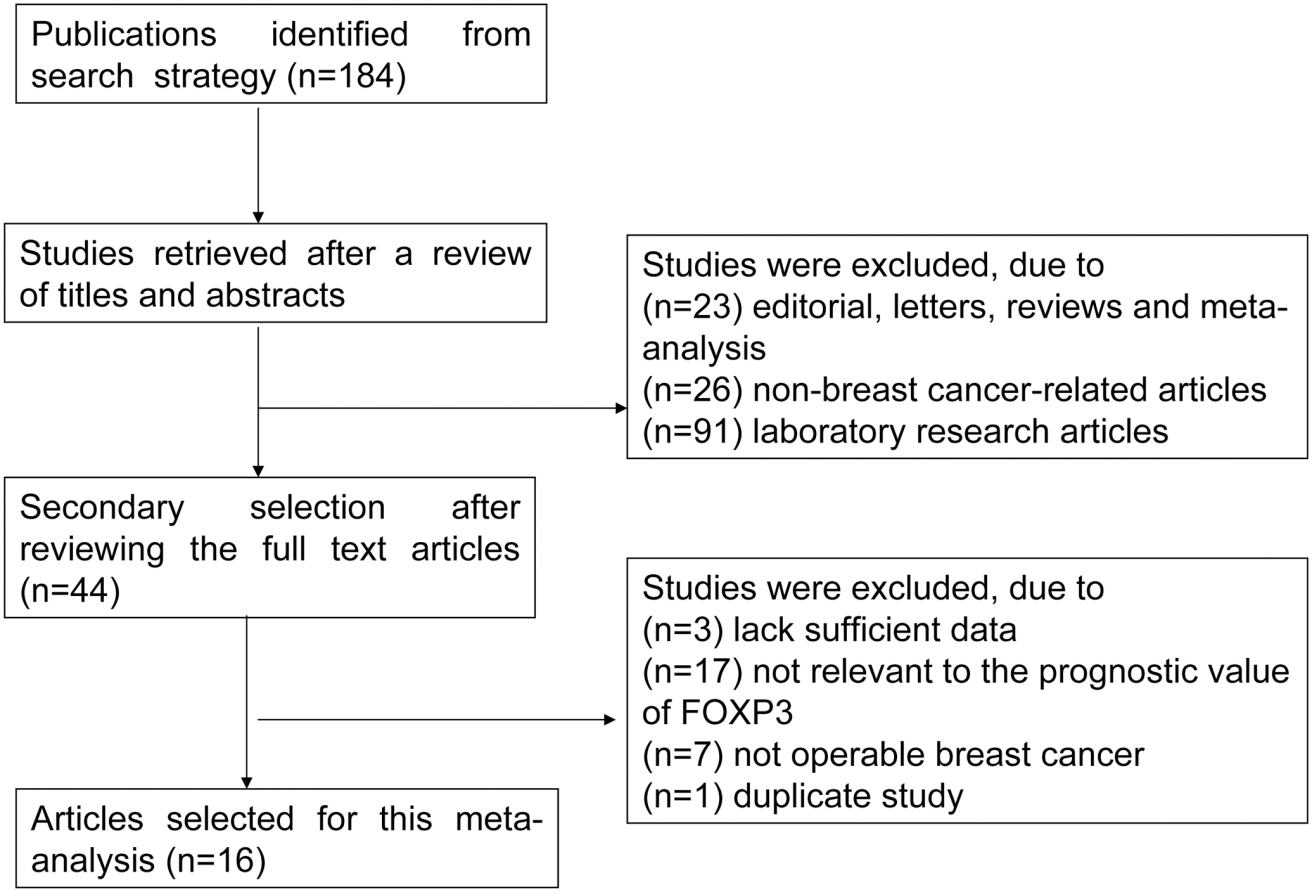
Meta-analysis flow chart.

A literature search of PubMed, EMBASE, the Cochrane Library and the annual meetings of ESMO and ASCO yielded 184 articles. After reviewing the titles and abstracts of these 184 articles, 140 articles were excluded by the inclusion criteria established for this study (see the Materials and Methods). Among the remaining articles whose full text was reviewed, 28 were excluded due to duplication, a lack of sufficient data, irrelevance to the prognostic value of FOXP3, or non-operable breast cancer. As a result, 16 publications were finally selected for a meta-analysis of the prognostic value of FOXP3 in BC (Fig 1). The detailed included and excluded studies are available in the S2 File. These publications provided data from a total of 13,217 patients who presented with BC, and the sample sizes ranged from 90 to 3,276 participants. Only Ali et al. [7] used data from previous studies conducted by Liu et al. [6], Mahmoud et al. [27], and other studies [28, 29]. Furthermore, among the 16 selected publications, DFS data were extracted from 9 of the articles, OS data were extracted from 14 of the articles, and clinicopathological parameters data were extracted from 8 of the articles. The details of the 16 included publications are summarized in Table 2. All of the data, except that from the study by Kim et al. [8], were used for univariate analysis. FOXP3 was expressed in intratumoral lymphocytes, peritumoral lymphocytes, and/or tumor cells. The FOXP3-positive cut-off values were based on either the median or on other values. The NOS scores of these studies ranged from 4 to 7 (with a mean of 5.38) (Table 2 and S1 Table), demonstrating that the quality of the included studies was acceptable.

**Table 2:** Main characteristics of the included studies.

First author [Ref no.]	Year of study	Country	Molecular subtype	Sample size (n)	Positive region	Cut-off value	Positive cases	Analysismethod	Study design	Quality score	Analysis index
Liu et al. [6]	2014	Canada	No specific	3276	IL	≥ 2	1031	UA	O	3+1+2	DFS, CP
Ali et al. [7]	2014	UK Canada	ER+	3263	IL	Positive	NP	UA	O and E	4+1+2	OS
Ali et al. [7]	2014	UK Canada	ER-	1391	IL	Positive	NP	UA	O and E	4+1+2	OS
Ali et al. [7]	2014	UK Canada	ER+	3263	PL	Positive	NP	UA	O and E	4+1+2	OS
Ali et al. [7]	2014	UK Canada	ER-	1391	PL	Positive	NP	UA	O and E	4+1+2	OS
Kim et al. [8]	2014	Korea	No specific	143	IL	Median	71	MA	O	2+1+1	OS, DFS
Kim et al. [8]	2014	Korea	No specific	143	PL	Median	71	MA	O	2+1+1	OS, DFS
Takenaka et al. [9]	2013	Japan	No specific	98	L	NP	56	UA	O	2+0+2	OS
Takenaka et al. [9]	2013	Japan	No specific	98	TC	NP	61	UA	O	2+0+2	OS
Takenaka et al. [9]	2013	Japan	No specific	100	TC	NP	57	UA	O	2+0+2	CP
Maeda et al. [10]	2013	Japan	No specific	90	L	Median Foxp3+ cell/TIL ratio	43	UA	O	3+1+1	OS, DFS
Sun et al. [11]	2014	China	No specific	208	IL	Median	104	UA	O	3+1+2	OS, DFS, CP
Won et al. [12]	2013	Korea	No specific	272	TC	Staining of ≥20% of cells	105	—	—	3+1+0	CP
Kim et al. [13]	2013	Korea	No specific	150	TC	Positive	18	UA	O	3+1+2	DFS, CP
West et al. [14]	2013	Canada	Triple negative	103	L	≥ 18 per mm^2^	NP	UA	O	3+1+2	OS
West et al. [14]	2013	Canada	ER-	175	L	≥ 18 per mm^2^	92	UA	O	3+1+2	DFS
Bates et al. [15]	2006	UK	No specific	237	L	Median (≥ 18 per mm^2^)	119	UA	O	3+1+2	OS, DFS
Bates et al. [15]	2006	UK	ER+	148	L	Median (≥ 15 per mm^2^)	60	UA	O	3+1+2	OS, DFS
Bates et al. [15]	2006	UK	ER-	77	L	Median (≥ 15 per mm^2^)	50	UA	O	3+1+2	OS, DFS
Droeser et al. [16]	2012	Switzerland	No specific	480	L	Total FOXP3+/CD4+ cells > 1	103	UA	O	2+1+2	OS
Ladoire et al. [17]	2012	France	No specific	1097	TC	Staining of ≥ 30% of TC	405	UA	E	3+1+2	OS, CP
Liu et al. [18]	2011	China	No specific	1270	IL	Median	646	UA	O	3+0+2	OS, DFS, CP
Liu et al. [18]	2011	China	ER+	778	IL	Median	272	UA	O	3+0+2	OS, DFS
Liu et al. [18]	2011	China	ER-	492	IL	Median	374	UA	O	3+0+2	OS, DFS
Liu et al. [18]	2011	China,	No specific	1270	PL	Median	NP	UA	O	3+0+2	OS, DFS
Yan et al. [19]	2011	Australia, UK	No specific	479	L	≥15 Treg per core	217	UA	O	3+0+2	OS
Yan et al. [19]	2011	Australia, UK	ER+	258	L	≥15 Treg per core	99	UA	O	3+0+2	OS
Merlo et al. [20] (Milan3)	2009	Italy	No specific	183	TC	Staining of ≥ 25% of cells	105	UA	E	3+0+3	OS, DFS, CP
Merlo et al. [20] (Milan1)	2009	Italy	No specific	214	TC	Staining of ≥ 25% of cells	156	UA	E	3+0+3	OS
Gobert et al. [21]	2009	France	No specific	184	IL	≥ 18	46	UA	O	2+1+2	OS
Gobert et al. [21]	2009	France	No specific	191	PL	48	46	UA	O	2+1+2	OS

IL: intratumoral lymphocytes; PL: peritunoral lymphocytes; TC: tumor cells; UK: United Kingdom; estrogen receptor; NP: not provided; UA: univariate analysis; MA: multivariate analysis; O: observational study; E: experimental study; OS: overallsurvival; DFS: disease-free survival; CP: clinicopathological parameters.

### Correlation of FOXP3 expression with clinicopathological data

Table 3 summarizes the pooled results of the correlations that were identified between FOXP3 expression and the clinicopathological features of BC. According to the FOXP3-positive regions, the examined studies were categorized into 3 subgroups, namely intratumoral lymphocytes, peritumoral lymphocytes, and/or a tumor cell subgroup. However, an analysis could only be performed if more than 2 studies were available for each subgroup. Accordingly, some clinicopathological features (such as progesterone receptor status, p53 mutation, Ki-67 index, lymphovascular invasion, local recurrence, distant metastasis, and advanced disease stage) could not be included in the analyses. For the tumor cells subgroup, no association between FOXP3 and any clinicopathological feature (including histological grade, tumor size, lymph node metastasis, ER status, and Her-2 status) was observed. However, for the intratumoral lymphocytes subgroup, the expression of FOXP3 was positively associated with histological grade (OR = 3.35; 95% CI: 2.09–5.35), lymph node metastasis (OR = 1.19; 95% CI: 1.06–1.34), and Her-2 expression (OR = 1.77; 95% CI: 1.52–2.07), while also being negatively associated with ER expression (OR = 0.30; 95% CI: 0.14–0.63). Furthermore, no significant correlation was observed between FOXP3 expression and the other clinicopathological features that were examined, such as patient age and tumor size.

**Table 3:** Main results for the meta-analysis between FOXP3 expression and clinicopathological parameters.

Positive region	Clinical parameters	Ref. No.	Overall OR(95% CI)	Heterogeneity test (Q, I2, P-value)	Model
Tumor cells	Histological grade (G3 vs. G1, G2)	12,13,17,20	1.20 (0.55–2.63)	28.64, 89.5%, 0.000	Random
	Tumor size (cm) (≥ 2 vs. < 2)	12,13,17,20	0.95 (0.77–1.17)	3.10, 3.10%, 0.377	Fixed
	Lymph nodes (N1 vs. N0)	12,13,20	1.31 (0.94–1.83)	2.59, 22.7%, 0.274	Fixed
	ER (ER+ vs. ER-)	12,13,20	0.57 (0.19–1.71)	19.99, 90.0%, 0.000	Random
	Her-2 (Her-2+ vs. Her-2-)	12,13,17,20	1.49 (0.68–3.26)	19.70, 84.8%, 0.000	Random
Intra-tumoral lymphocytes	Patient age (≥ 50 y vs. < 50 y)	6,9,18	0.80 (0.49–1.31)	17.66, 88.7%, 0.000	Random
	Histological grade (G3 vs. G1, G2)	6,9,11,18	3.35 (2.09–5.35)	15.94, 81.2%, 0.001	Random
	Tumor size (cm) (≥ 2 vs. < 2)	6,9,11,18	1.09 (0.96–1.23)	0.82, 0.0%, 0.845	Fixed
	Lymph nodes (N1 vs. N0)	6,9,11,18	1.19 (1.06–1.34)	2.95, 0.0%, 0.399	Fixed
	ER (ER+ vs. ER-)	6,9,11,18	0.30 (0.14–0.63)	57.0, 94.7%, 0.000	Random
	Her-2 (Her-2+ vs. Her-2-)	6,9,11,18	1.77 (1.52–2.07)	1.63, 0.0%, 0.653	Fixed

OR, odds ratio; CI, confidence interval; Q, heterogeneity Chi-squared; I^2^, I-squared; ER, estrogen receptor.

### FOXP3 expression and DFS

**Fig 2:**
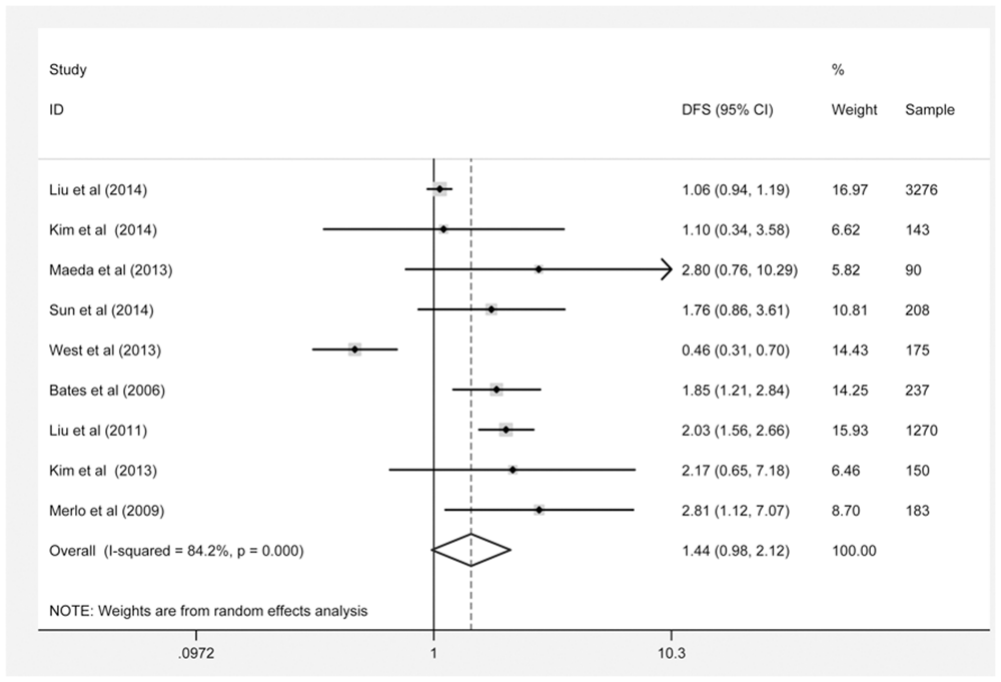
FOXP3 expression and DFS.

**Fig 3:**
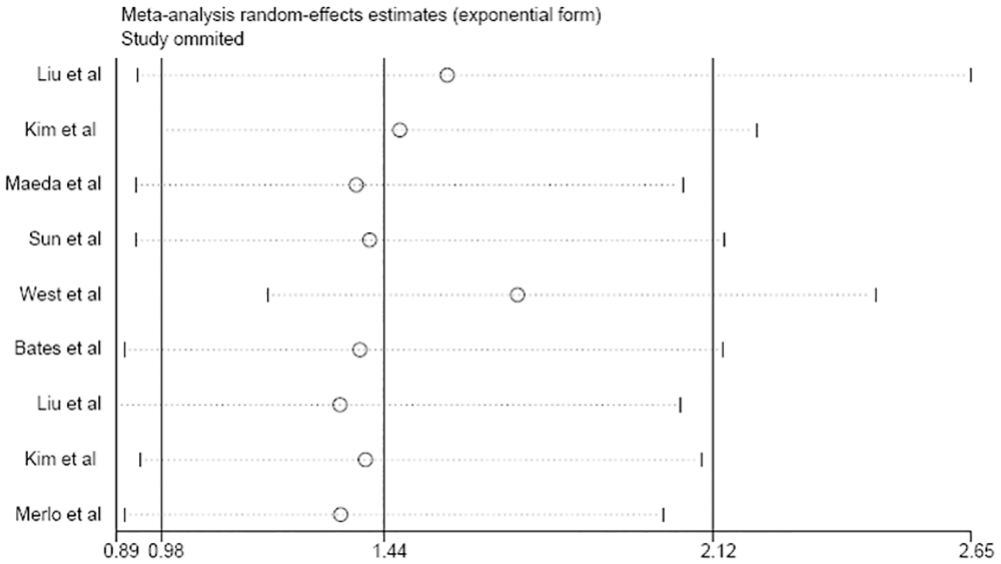
Sensitivity analysis of DFS in the meta-analysis.

The pooled HR values showed that high levels of FOXP3 expression were not significantly associated with DFS in relation to BC (HR: 1.44, 95% CI: 0.98–2.12) (Fig 2). In addition, significant heterogeneity (P = 0.000, I^2^ = 84.2%) was observed when the pooled HR value for DFS was analyzed using a random-effects model. In a sensitivity analysis, excluding the study by West et al. [14] caused the pooled HR to shift to 1.72 (95% CI: 1.20–2.46) and decreased heterogeneity (P = 0.000, I^2^ = 76.4%). When the study by Kim et al. [8] (the only study with multivariate analysis) was omitted, the pooled HR was not substantially changed (Fig 3). To minimize heterogeneity, the subgroup analyses were performed according to FOXP3-positive region, ER status, sample size, study design, geographic region and FOXP3-positive cut-off value. When stratifying for FOXP3-positive region, the pooled HR for DFS was 2.55 (95% CI: 1.23–5.30) in tumor cells, 1.46 (95% CI: 0.91–2.35) in intratumoral lymphocytes, and 1.57 (95% CI: 0.48–5.12) in peritumoral lymphocytes. In the subgroup analysis by ER status, a stronger association was observed along with no heterogeneity in the ER+ subgroup (summary HR: 1.83, 95% CI: 1.36–2.47), while the ER- subgroup exhibited no association. We also observed a significant correlation in studies from Asian regions (HR: 1.98, 95% CI: 1.56–2.50) and in studies using the median as the FOXP3-positive cut-off value (HR: 1.94, 95% CI: 1.57–2.39). However, we did not observe any correlations in the other subgroup analyses based on sample size and study design (Table 4).

**Table 4:** Association between FOXP3 expression and DFS stratified according to FOXP3-positive region, ER status, sample size, study design, geographic region and the FOXP3-positive cut-off value.

Stratified analysis		Ref. no.	HR (95% CI)	P-Value	HeterogeneityI2 P-value	
**Positive region**	Intratumoral lymphocytes	6, 8, 11, 18	1.46 (0.91–2.35)	0.120	85.2%	0.000
	Tumor cells	13, 20	2.55 (1.23–5.30)	0.012	0.0%	0.738
	Peritumoral lymphoctes	8, 18	1.57 (0.48–5.12)	0.455	73.8%	0.051
**ER status**	Positive	15, 18	1.83 (1.36–2.47)	0.000	0.0%	0.445
	Negative	14, 15, 18	0.86 (0.40–1.87)	0.710	85.1%	0.001
**Sample size (n)**	>300	6, 18	1.45 (0.77–2.74)	0.252	94.8%	0.000
	<300	8, 10, 11, 13, 14, 15, 20	1.52 (0.80–2.88)	0.203	81.0%	0.000
**Study design**	Observatinal study	6, 8, 10, 11, 13, 14, 15, 18	1.35 (0.91–2.02)	0.140	85.2%	0,000
	Others	20	ˉ	ˉ	ˉ	ˉ
**Geographic region**	Asian	8, 10, 11, 13, 18	1.98 (1.56–2.50)	0.000	0.0%	0.846
	Not Asian	6, 14, 15, 20	1.16 (0.66–2.05)	0.606	88.7%	0.000
**Cut-off value**	Median	8, 10, 11, 15, 18	1.94 (1.57–2.39)	0.000	0.0%	0.840
	Not Median	6, 13, 14, 20	1.13 (0.59–2.16)	0.720	85.7%	0.000

DFS, disease-free survival; ER, estrogen receptor; HR, hazard ratio; CI, confidence interval; I^2^, I-squared.

### FOXP3 expression and OS

**Fig 4:**
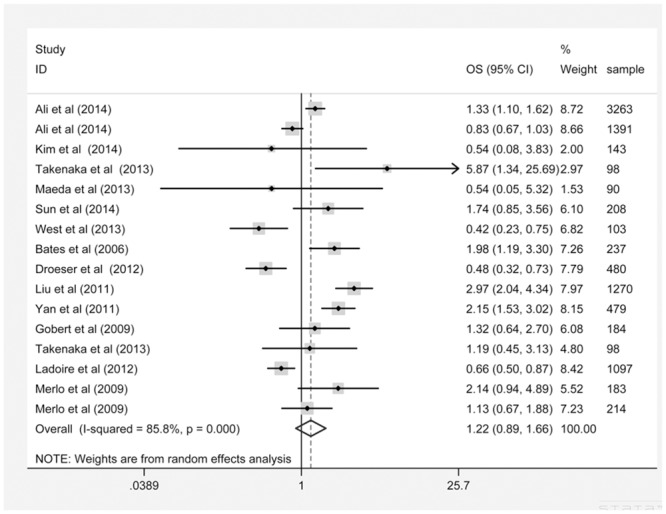
FOXP3 expression and OS.

**Fig 5:**
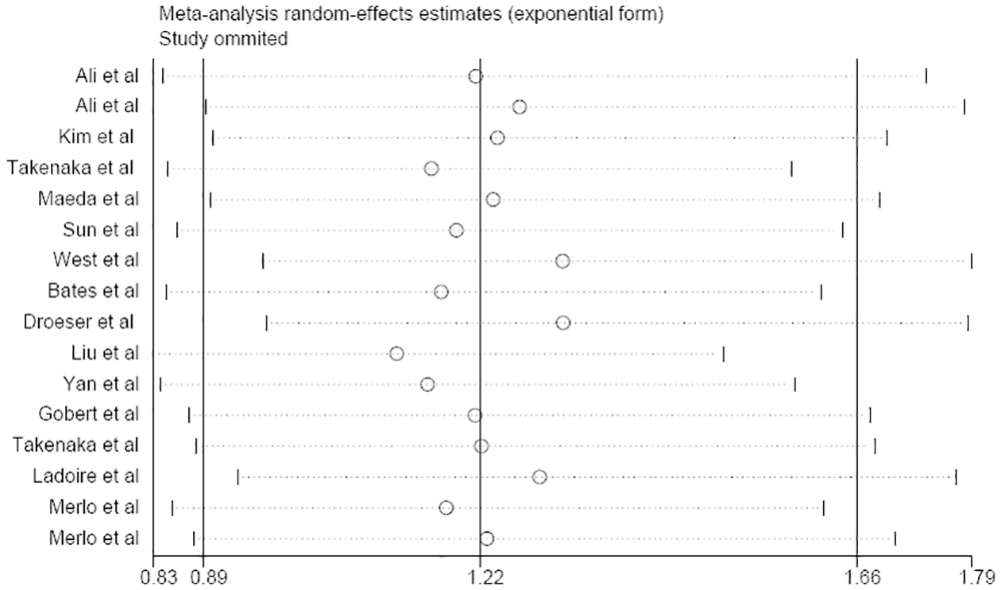
Sensitivity analysis of OS in the meta-analysis.

No significant correlation between FOXP3 expression and patient OS was observed (HR: 1.22, 95% CI: 0.89–1.66) in a random-effects model with significant heterogeneity (P = 0.000, I^2^ = 85.8%) (Fig 4). Sensitivity analysis showed that the pooled estimate of the effect of FOXP3 expression on the OS of BC patients did not vary substantially with the exclusion of any one study, demonstrating that the results of this meta-analysis are stable (Fig 5). Subgroup analyses revealed that the ER status, the geographic region and the FOXP3-positive cut-off value significantly influenced the pooled HR result. We observed a significant correlation for the studies in the ER+ subgroup (HR: 1.87, 95% CI: 1.28–2.73), studies in the Asian region (HR: 1.93, 95% CI: 1.12–3.35) and studies that used the median as the cut-off value (HR: 2.06, 95% CI: 1.36–3.11). However, subgroup analysis based on other factors such as staining pattern, sample size and study design did not significantly influence the pooled HR results (Table 5).

**Table 5:** Association between FOXP3 expression and OS stratified according to FOXP3-positive region, ER status, sample size, study design, geographic region and the FOXP3-positive cut-off value.

Stratified analysis		Ref. no.	HR (95% CI)	P-Value	Heterogeneity I2 P-value	
**Positive region**	Intratumoral lymphocytes	7, 8, 11, 18, 21	1.41 (0.91–2.19)	0.127	86.1%	0.000
	Tumor cells	9, 17, 20	1.07 (0.64–1.80)	0.801	68.8%	0.022
	Peritumoral lymphoctes	7, 8, 18, 21	1.05 (0.91–1.22)	0.497	8.1%	0.360
**ER status**	Positive	7, 15, 18, 19	1.87 (1.28–2.73)	0.001	60.0%	0.057
	Negative	7, 14, 15, 18	0.82 (0.53–1.28)	0.386	64.1%	0.039
**Sample size (n)**	>300	7, 16, 17, 18, 19	1.14 (0.72–1.81)	0.572	93.8%	0.000
	<300	8, 9, 10, 11, 14, 15, 20, 21	1.30 (0.84–2.00)	0.242	63.4%	0.003
**Study design**	Observational study	8, 9, 10, 11, 14, 15, 16, 18, 19, 21	1.31 (0.78–2.20)	0.300	85.9%	0.000
	Others	7, 17, 20	1.03 (0.73–1.45)	0.864	82.9%	0.000
**Geographic region**	Asian	8, 9, 10, 11, 18	1.93 (1.12–3.35)	0.019	46.4%	0.097
	Not Asian	7, 14, 15, 16, 17, 19, 20,21	1.05 (0.76–1.45)	0.782	87.3%	0.000
**Cut-off value**	Median	8, 10, 11, 15, 18	2.06 (1.36–3.11)	0.001	37.2%	0.173
	Not Median	7, 9, 14, 16, 17, 19, 20, 21	1.05 (0.76–1.46)	0.751	85.7%	0.000

OS, overall survival; ER, estrogen receptor; HR, hazard ratio; CI, confidence interval; I^2^, I-squared.

### Publication bias

**Fig 6:**
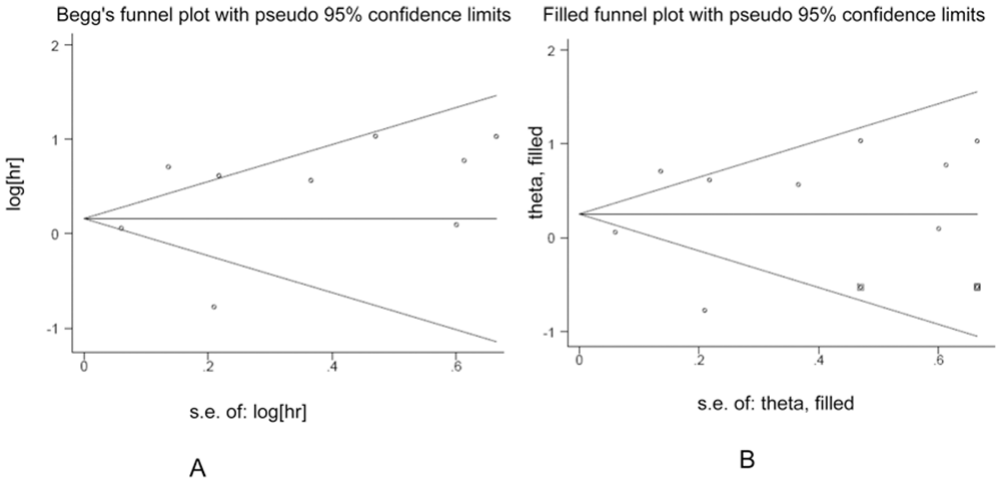
Funnel plots in the context of DFS without and with trim and fill. The pseudo 95% CI is computed as part of the analysis that produces the funnel plot, and corresponds to the expected 95% CI for a given SE.

**Fig 7:**
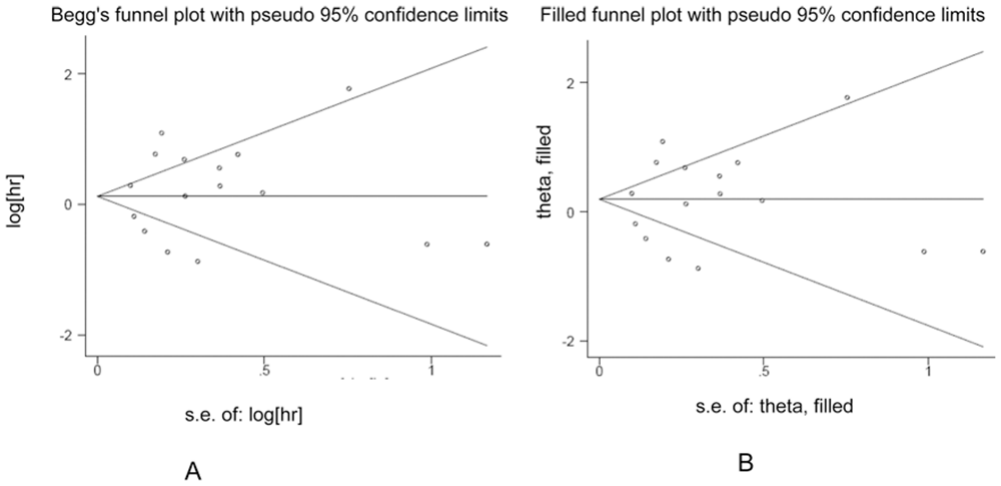
Funnel plots in the context of OS without and with trim and fill. The pseudo 95% CI is computed as part of the analysis that produces the funnel plot, and corresponds to the expected 95% CI for a given SE.

For DFS, the shape of the funnel plots appeared asymmetrical, which might indicate a publication bias. However, Begg’s and Egger’s tests showed a statistically non-significant value (P = 1.000 and 0.362, respectively) (Fig 6A). Then we conducted sensitivity analysis using the trim and fill method. After imputing two hypothetical studies, the funnel plots become symmetrical (Fig 6B) and the pooled analysis did not change significantly (HR: 1.29, 95% CI: 0.90–1.84). With respect to the OS, no evidence for asymmetry was observed in the funnel plots, and Begg’s and Egger’s tests also revealed no evidence of publication bias (P = 0.822 and 0.658, respectively) (Fig 7A). Moreover, the trim and fill method showed that the theoretical missing studies didn’t exist (Fig 7B).

## Discussion

To the best of our knowledge, this is the first large-scale meta-analysis to systematically investigate the prognostic role of FOXP3 expression in the context of BC. High FOXP3 expression levels did not significantly correlate with DFS and OS with significant heterogeneity. These results imply that several factors may have influenced the pooled results. When further subgroup analyses were conducted, categorized by positive region, ER status, geographical region, and FOXP3-positive cut-off value, FOXP3 expression exhibited a significant prognostic role.

Tumor cells could recruit lymphocytes infiltrating into tumor microenvironment and trigger an immune response in the host. These cells, also named tumor infiltrating lymphocytes (TILs), are composed of different kind of lymphocytes, including CD4+ and CD8+ T cells, Tregs, B cells, NK cells and NKT cells. CD4+ and CD8+ T cells could exert antitumor function. Intratumoral Tregs, defined as Tregs in the tumor bed, could regulate the function of many other immune cells. Therefore, different infiltrating lymphocytes may play different role on tumor initiation and progression [30]. In a recent meta-analysis including studies in solid tumors of any kind, the results showed that CD4+ TILs and CD8+ TILs were both associated with an improved overall survival. However, Tregs were not linked to DFS and OS with significant heterogeneity, implying that the prognositic role of Tregs may depend on the different tumor type, or other clinicopathological parameters [31]. In many solid tumors such as Hodgkin lymphoma, melanoma, gastric and ovarian carcinoma, Tregs are associated with a poor prognosis. However, in other tumors such as head and neck cancer, colorectal and bladder cancer, Tregs are a biomarker of good clinical outcome [32]. Some studies tried to figure out the mechanisms for the dual role of Tregs. On the one hand, Tregs can induce immune tolerance and lead to tumor progression by the following mechanisms: secretion of immunosuppressive molecules such as transforming growth factor beta (TGFβ), IL-10, and CCL22; directly cytolysis of NK cells and CD8+ cells; metabolic disruption; and promoting angiogenesis [1, 33–35]. On the other hand, Tregs can inhibit tumor-promoting inflammation induced by bacteria infection, thereby contributing to an improved outcome [36]. Another prevalent theory was proposed by Nishikawa et al. [4]. Based on their theory, FOXP3+ T cells could be classified into three subpopulations according to the expression levels of FOXP3 and the cell surface molecule CD45RA: naive or resting Treg cells (Fr. I), effector Treg cells (Fr. II), and non-Treg cells (Fr. III). These subpopulations of infiltrating FOXP3+ T cells have different functions. And the predominant compositions of FOXP3+ T cells in tumor tissues are quite different, thereby leading to different prognoses [4]. The present meta-analysis demonstrates that in the context of BC, intratumoral FOXP3+ Tregs positively correlated with adverse clinicopathological parameters such as histological grade, lymph node metastasis, ER- status, and Her-2+ status. However, intratumoral FOXP3+ Tregs were not associated with DFS and OS. This relationship between FOXP3 expression and pathological finding is consistent with the findings of Ladoire et al. [37], who observed that patients who achieved a pathologic complete response after chemotherapy exhibited decreased levels of FOXP3+ Treg cells. Besides, this finding implies that even in a definite tumor type, intratumoral FOXP3+ Tregs may possess anti-tumor activity, in addition to their tumorigenic properties.

For tumor cells, the pooled results showed that FOXP3 was significantly associated with DFS but was not associated with clinicopathological parameters or OS with significant heterogeneity. In a study by Ladoire et al. [17], FOXP3 expression was associated with better OS; however, in a study by Merlo et al. [20], FOXP3 expression was associated with poor OS. This discrepancy may be due to differences in the populations that were studied in these reports, including differences in country, race, and disease stage. Another explanation is that the distribution of FOXP3 expression may differ between the cytoplasm and the nucleus. Takenaka et al. [9] have further suggested that cytoplasmic and nuclear FOXP3 have different prognostic roles, which may be influenced by the posttranscriptional modifications of FOXP3. However, the underlying mechanism(s) require further elucidation, particularly with respect to the possibility that FOXP3 may mediate tumor-suppressive versus tumor-promoting activities. For example, in gastric cancer, FOXP3 acts as a tumor suppressor protein by inhibiting the activity of NF-κB and by interfering with the expression of COX2 [38]. Correspondingly, Ma et al. [39] found that high levels of FOXP3 expression in gastric cancer cells predict better survival. In melanoma cells, Tan et al. [40] demonstrated that FOXP3 expression suppresses cell proliferation, increases cell differentiation and apoptosis, and reduces tumorigenesis. In contrast, downregulating FOXP3 expression in thyroid cancer resulted in the downregulation of NF-κB subunit p65 and cyclin D1 concomitantly with the upregulation of caspase-3 levels. Taken together, these changes lead to decreased cell proliferation and migration and increased levels of apoptosis [41]. Moreover, in head and neck cancer, FOXP3 may cooperate with the inflammation factor COX2 and with the migration/invasion factors AHNAK and cortactin to promote tumor progression [42]. Thus, we hypothesize that the signaling pathway networks downstream of FOXP3 are critical for the complicated functions mediated by FOXP3 in tumor cells. Further investigation is also needed to explore what controls the activation or inhibition of these signaling pathways in response to FOXP3.

According to the St. Gallen International Expert Consensus, ER+ subtype breast cancer can be additionally classified into three molecular subtypes: Luminal A (HER2 -; Ki-67 low, <14%), Luminal B (HER2-, Ki-67-high) and Luminal B (HER2 overexpressed or amplified). Adjuvant endocrine therapy after radical mastectomy is an essential treatment for each of these ER+ subtypes. Because the Luminal B (HER2-, Ki-67 high) subtype and Luminal B (HER2 overexpressed or amplified) subtype harbor a high risk for recurrence and metastasis, other treatment modalities, such as chemotherapy and anti-HER2 therapy, are recommended [43]. In the current study, we uncovered a relationship between the expression of FOXP3 in intratumoral lymphocytes and adverse clinicopathological parameters, such as high histological grade, lymph node metastasis, and Her-2 expression. In addition, we showed that FOXP3 expression in the ER+ subgroup showed poor DFS and OS. Therefore, we suggest that for ER+ subgroup patients with FOXP3 expression, chemotherapy combined with endocrine therapy is rational. Further studies are needed to investigate whether FOXP3 can be used as a therapeutic target in the ER+ subgroup to improve clinical outcomes. Until now, limited data are available concerning the underlying mechanisms of the unfavorable prognostic role for FOXP3 expression in ER+ subgroup. Estrogen has been reported to promote the proliferation of the FOXP3 Treg cells and boost the suppressive function of these cells. Additional experiment showed that FOXP3+ Treg cells may express ER [44]. As mentioned above, there are various subpopulations of FOXP3+ T cells. We hypothesized that in ER+ subgroup patients, the predominant subpopulations of FOXP3+ T cells was hormonal dependent, and may possess anti-tumor activity. Therefore, FOXP3+ Treg cells in ER+ subgroup patients correlated with an unfavorable prognosis. Further research is needed to confirm this hypothesis.

Notably, FOXP3 expression was not correlated with survival in ER- breast cancers, indicating that FOXP3 protein may play a different role depending on ER status. Few studies have addressed the exact molecular mechanisms involved. However, recent results suggest that breast cancers may have different CD8+ CTL counts according to their ER status, which may affect the prognostic role of FOXP3 [6]. Additionally, a study by Kim et al. [45] showed that ER expression correlated with an increased FOXP3(+) Treg/CD4(+) T-cell ratio but not with an increased number of FOXP3(+) cells. These results imply that breast cancers characterized by different ER states may incur different immune responses and thus may lead to different clinical outcomes.

Moreover, subgroup analyses with other factors were performed to identify other sources of heterogeneity. We showed that FOXP3 expression was correlated with poor DFS and OS in the Asian region but not in other regions. This discrepancy may be due to different genetic backgrounds, surgical procedures, and chemotherapy agents. Moreover, we noted that the cut-off value for FOXP3 expression varied in different studies, and this factor may contribute to heterogeneity. Additional subgroup analysis suggested that FOXP3 expression was associated with poor DFS and OS in studies using the median as the FOXP3-positive cut-off value, yet this association was not observed for studies using other cut-off values. This result indicates that FOXP3 may play a dual role in tumor progression, displaying either anti-tumor or tumorigenic activities. When FOXP3 expression was above the median, it was consistently associated with an unfavorable prognosis. Therefore, additional studies using standardized technical protocols are needed in order to diminish heterogeneity. Additionally, sensitivity analysis for DFS identified an extreme study, the study by West et al. [14], which accounted for 14.43% of the sample size. This study demonstrated that high levels of FOXP3 expression represented a marker of good prognosis. Together with subgroup analysis, we propose that several characteristics may have contributed to this result, such as ER- breast cancer, a non-Asian study region and the use of FOXP3-positive cut-off values other than the median. Subgroup analysis by other factors, such as sample size and study design, did not significantly influence the pooled results of DFS and OS.

The present study had several limitations. Firstly, although this meta-analysis included a large population, the number of studies included in the subgroup analyses was relatively small. Notably, only a few of the selected studies focused on the prognostic role of FOXP3 expression distribution in tumor cells. Secondly, with respect to DFS, we generated asymmetrical funnel plots, suggesting a potential publication bias. However, the trim and fill sensitivity analysis showed that publication bias may have little effect on the pooled results. Thirdly, there is limit prognostic information stratified by tumor stage, progesterone receptor status and treatment modality. And this meta-analysis was based on data abstracted from publications, instead of individual patient data. Therefore, we could not conduct subgroup analyses for these parameters. Fourthly, the quality scales of some studies were still not so high, which may potentially affect the pooled results.

In conclusion, the results of this meta-analysis indicate that the prognostic role of FOXP3 expression in operable breast cancer cases depends on the FOXP3-positive region, the ER status, the geographic region and the FOXP3-positive cut-off value.

## Supporting Information

S1 File The detailed search strategies in the main databases. (DOC)

S2 File The full list of the detailed liturature included and excluded. (DOC)

S3 File PRISMA Checklist. (DOC)

S1 Table The NOS scores of the included studies. (Note: Data given as the definite option of each item. ^※^ means that the score was zero.)
(DOCX)
