# Supplementary material for: The Prognostic Value of Forkhead Box P3 Expression in Operable Breast Cancer: A Large-Scale Meta-Analysis
Source: PLoS One. 2015 Aug 25;10(8):e0136374. doi: 10.1371/journal.pone.0136374 (PMC4549287; doi:10.1371/journal.pone.0136374)
Supplement: S1 File — (DOC) [file pone.0136374.s001.doc]

**S1 File. The detailed search strategies in the main databases.**

**MEDLINE search strategy**

1.mammar* and (neoplasm* or cancer* or tumour* or tumor* or carcinoma* or adenocarcinoma* or sarcoma* or dcis or ductal or infiltrat* or intraduct* or lobular or medullary) Field: Title/Abstract

2.breast and (neoplasm* or cancer* or tumour* or tumor* or carcinoma* or adenocarcinoma* or sarcoma* or dcis or ductal or infiltrat* or intraduct* or lobular* or medullary*) Field: Title/Abstract

3.“Breast Neoplasms”[MeSH]

4.“Neoplasms, Glandular and Epithelial”[MeSH]

5. 1 or 2 or 3 or 4

6. prognos* or outcome* or progress or metasta* or relapse* or recurren* or surviv* or death* or die* or dead or dying or mortality.

7. “prognosis” [MeSH]

8. 6 or 7

9. FOXP3 protein, human [MeSH]

10.SCURFIN or IPEX or forkhead box P3 protein

11. 9 or 10

12. 5 and 8 and 11

**EMBASE search strategy**

#14 #7 AND #10 AND #13

#13. #11 OR #12

#12.prognos$ or outcome$ or progress or metasta$ or relapse$ or recurren$ or surviv$ or death$ or die$ or dead or dying or mortality

#11. 'prognosis'/exp

#10. #8 OR #9

#9. SCURFIN or IPEX or forkhead box P3 protein

#8. 'FOXP3 protein'/exp

#7. #1 OR #2 OR #3 OR #4 OR #5 OR #6

#6.mammar$ and (neoplasm$ or cancer$ or tumour$ or tumor$ or carcinoma$ or adenocarcinoma$ or sarcoma$ or dcis or ductal or infiltrat$ or intraduct$ or lobular or medullary).

#5.breast and (neoplasm$ or cancer$ or tumour$ or tumor$ or carcinoma$ or adenocarcinoma$ or sarcoma$ or dcis or ductal or infiltrat$ or intraduct$ or lobular$ or medullary$).

#4.neoplasms, glandular and epithelial

#3.Breast Tumor

#2..Breast Carcinoma

#1.'Breast Disease'/exp
