# Supplementary material for: The Prognostic Value of Forkhead Box P3 Expression in Operable Breast Cancer: A Large-Scale Meta-Analysis
Source: PLoS One. 2015 Aug 25;10(8):e0136374. doi: 10.1371/journal.pone.0136374 (PMC4549287; doi:10.1371/journal.pone.0136374)
Supplement: S2 File — (DOC) [file pone.0136374.s002.doc]

**S2 File. The full list of the detailed liturature included and excluded**

**Included studies**

1. Ali HR, Provenzano E, Dawson SJ*, et al*: Association between CD8+ T-cell infiltration and breast cancer survival in 12,439 patients. Annals of oncology : official journal of the European Society for Medical Oncology / ESMO 25: 1536-1543, 2014.

2. Bates GJ, Fox SB, Han C*, et al*: Quantification of regulatory T cells enables the identification of high-risk breast cancer patients and those at risk of late relapse. Journal of clinical oncology : official journal of the American Society of Clinical Oncology 24: 5373-5380, 2006.

3. Droeser R, Zlobec I, Kilic E*, et al*: Differential pattern and prognostic significance of CD4+, FOXP3+ and IL-17+ tumor infiltrating lymphocytes in ductal and lobular breast cancers. BMC cancer 12: 134, 2012.

4. Gobert M, Treilleux I, Bendriss-Vermare N*, et al*: Regulatory T cells recruited through CCL22/CCR4 are selectively activated in lymphoid infiltrates surrounding primary breast tumors and lead to an adverse clinical outcome. Cancer research 69: 2000-2009, 2009.

5. Kim MH, Koo JS and Lee S: FOXP3 expression is related to high Ki-67 index and poor prognosis in lymph node-positive breast cancer patients. Oncology 85: 128-136, 2013.

6. Kim S, Lee A, Lim W*, et al*: Zonal difference and prognostic significance of foxp3 regulatory T cell infiltration in breast cancer. Journal of breast cancer 17: 8-17, 2014.

7. Ladoire S, Mignot G, Dalban C*, et al*: FOXP3 expression in cancer cells and anthracyclines efficacy in patients with primary breast cancer treated with adjuvant chemotherapy in the phase III UNICANCER-PACS 01 trial. Annals of oncology : official journal of the European Society for Medical Oncology / ESMO 23: 2552-2561, 2012.

8. Liu F, Lang R, Zhao J*, et al*: CD8(+) cytotoxic T cell and FOXP3(+) regulatory T cell infiltration in relation to breast cancer survival and molecular subtypes. Breast cancer research and treatment 130: 645-655, 2011.

9. Liu S, Foulkes WD, Leung S*, et al*: Prognostic significance of FOXP3+ tumor-infiltrating lymphocytes in breast cancer depends on estrogen receptor and human epidermal growth factor receptor-2 expression status and concurrent cytotoxic T-cell infiltration. Breast cancer research : BCR 16: 432, 2014.

10. Maeda N, Yoshimura K, Yamamoto S*, et al*: Expression of B7-H3, a potential factor of tumor immune evasion in combination with the number of regulatory T cells, affects against recurrence-free survival in breast cancer patients. Annals of surgical oncology 21 Suppl 4: S546-554, 2014.

11. Merlo A, Casalini P, Carcangiu ML*, et al*: FOXP3 expression and overall survival in breast cancer. Journal of clinical oncology : official journal of the American Society of Clinical Oncology 27: 1746-1752, 2009.

12. Sun S, Fei X, Mao Y*, et al*: PD-1(+) immune cell infiltration inversely correlates with survival of operable breast cancer patients. Cancer immunology, immunotherapy : CII 63: 395-406, 2014.

13. Takenaka M, Seki N, Toh U*, et al*: FOXP3 expression in tumor cells and tumor-infiltrating lymphocytes is associated with breast cancer prognosis. Molecular and clinical oncology 1: 625-632, 2013.

14. West NR, Kost SE, Martin SD*, et al*: Tumour-infiltrating FOXP3(+) lymphocytes are associated with cytotoxic immune responses and good clinical outcome in oestrogen receptor-negative breast cancer. British journal of cancer 108: 155-162, 2013.

15. Won KY, Kim HS, Sung JY*, et al*: Tumoral FOXP3 has potential oncogenic function in conjunction with the p53 tumor suppressor protein and infiltrated Tregs in human breast carcinomas. Pathology, research and practice 209: 767-773, 2013.

16. Yan M, Jene N, Byrne D*, et al*: Recruitment of regulatory T cells is correlated with hypoxia-induced CXCR4 expression, and is associated with poor prognosis in basal-like breast cancers. Breast cancer research : BCR 13: R47, 2011.

**Excluded studies**

**Editorial, letters, reviews and meta-analysis**

1. Banin Hirata BK, Oda JM, Losi Guembarovski R, Ariza CB, de Oliveira CE and Watanabe MA: Molecular markers for breast cancer: prediction on tumor behavior. Disease markers 2014: 513158, 2014.

2. Douglass S, Ali S, Meeson AP, Browell D and Kirby JA: The role of FOXP3 in the development and metastatic spread of breast cancer. Cancer metastasis reviews 31: 843-854, 2012.

3. Elton TS, Selemon H, Elton SM and Parinandi NL: Regulation of the MIR155 host gene in physiological and pathological processes. Gene 532: 1-12, 2013.

4. Ferretti G: Forkhead box P3-positive regulatory T cells as therapeutic target for breast cancer. Journal of clinical oncology : official journal of the American Society of Clinical Oncology 25: e29; author reply e30, 2007.

5. Fulton A, Miller F, Weise A and Wei WZ: Prospects of controlling breast cancer metastasis by immune intervention. Breast disease 26: 115-127, 2006.

6. Jiang LL and Ruan LW: Association between FOXP3 promoter polymorphisms and cancer risk: A meta-analysis. Oncology letters 8: 2795-2799, 2014.

7. Katoh H, Zheng P and Liu Y: Signalling through FOXP3 as an X-linked tumor suppressor. The international journal of biochemistry & cell biology 42: 1784-1787, 2010.

8. Katoh H, Zheng P and Liu Y: FOXP3: genetic and epigenetic implications for autoimmunity. Journal of autoimmunity 41: 72-78, 2013.

9. Lal A, Chan L, Devries S*, et al*: FOXP3-positive regulatory T lymphocytes and epithelial FOXP3 expression in synchronous normal, ductal carcinoma in situ, and invasive cancer of the breast. Breast cancer research and treatment 139: 381-390, 2013.

10. Linehan DC and Goedegebuure PS: CD25+ CD4+ regulatory T-cells in cancer. Immunologic research 32: 155-168, 2005.

11. Liu Y and Zheng P: FOXP3 and breast cancer: implications for therapy and diagnosis. Pharmacogenomics 8: 1485-1487, 2007.

12. Lu H: FOXP3 expression and prognosis: role of both the tumor and T cells. Journal of clinical oncology : official journal of the American Society of Clinical Oncology 27: 1735-1736, 2009.

13. Mao Y, Qu Q, Zhang Y, Liu J, Chen X and Shen K: The value of tumor infiltrating lymphocytes (TILs) for predicting response to neoadjuvant chemotherapy in breast cancer: a systematic review and meta-analysis. PloS one 9: e115103, 2014.

14. Medema RH and Burgering BM: The X factor: skewing X inactivation towards cancer. Cell 129: 1253-1254, 2007.

15. Mir R, Pradhan SJ and Galande S: Chromatin organizer SATB1 as a novel molecular target for cancer therapy. Current drug targets 13: 1603-1615, 2012.

16. Oda JM, Hirata BK, Guembarovski RL and Watanabe MA: Genetic polymorphism in FOXP3 gene: imbalance in regulatory T-cell role and development of human diseases. Journal of genetics 92: 163-171, 2013.

17. Rech AJ and Vonderheide RH: Clinical use of anti-CD25 antibody daclizumab to enhance immune responses to tumor antigen vaccination by targeting regulatory T cells. Annals of the New York Academy of Sciences 1174: 99-106, 2009.

18. Tsukasaki K and Tobinai K: Human T-cell lymphotropic virus type I-associated adult T-cell leukemia-lymphoma: new directions in clinical research. Clinical cancer research : an official journal of the American Association for Cancer Research 20: 5217-5225, 2014.

19. Wang L, Liu R, Ribick M, Zheng P and Liu Y: FOXP3 as an X-linked tumor suppressor. Discovery medicine 10: 322-328, 2010.

20. Watanabe MA, Oda JM, Amarante MK and Cesar Voltarelli J: Regulatory T cells and breast cancer: implications for immunopathogenesis. Cancer metastasis reviews 29: 569-579, 2010.

21. Wolf AM, Rumpold H, Wolf D*, et al*: Role of forkhead box protein 3 expression in invasive breast cancer. Journal of clinical oncology : official journal of the American Society of Clinical Oncology 25: 4499-4500; author reply 4500-4491, 2007.

22. Wolf D, Wolf AM and Tzankov A: Comment on "Cutting edge: depletion of Foxp3+ cells leads to induction of autoimmunity by specific ablation of regulatory T cells in genetically targeted mice". Journal of immunology (Baltimore, Md : 1950) 184: 4051, 2010.

23. Zlobec I and Lugli A: Invasive front of colorectal cancer: dynamic interface of pro-/anti-tumor factors. World journal of gastroenterology : WJG 15: 5898-5906, 2009.

**Non-breast cancer-related articles**

1. Azimi Mohamadabadi M, Hassan ZM, Zavaran Hosseini A*, et al*: Arteether exerts antitumor activity and reduces CD4+CD25+FOXP3+ T-reg cells in vivo. Iranian journal of immunology : IJI 10: 139-149, 2013.

2. Bose A, Chakraborty T, Chakraborty K, Pal S and Baral R: Dysregulation in immune functions is reflected in tumor cell cytotoxicity by peripheral blood mononuclear cells from head and neck squamous cell carcinoma patients. Cancer immunity 8: 10, 2008.

3. Brown CY, Sadlon T, Gargett T*, et al*: Robust, reversible gene knockdown using a single lentiviral short hairpin RNA vector. Human gene therapy 21: 1005-1017, 2010.

4. Bushel PR, McGovern R, Liu L*, et al*: Population differences in transcript-regulator expression quantitative trait loci. PloS one 7: e34286, 2012.

5. Chen X, Hamano R, Subleski JJ, Hurwitz AA, Howard OM and Oppenheim JJ: Expression of costimulatory TNFR2 induces resistance of CD4+FoxP3- conventional T cells to suppression by CD4+FoxP3+ regulatory T cells. Journal of immunology (Baltimore, Md : 1950) 185: 174-182, 2010.

6. Dang Y, Knutson KL, Goodell V*, et al*: Tumor antigen-specific T-cell expansion is greatly facilitated by in vivo priming. Clinical cancer research : an official journal of the American Association for Cancer Research 13: 1883-1891, 2007.

7. Heinze E, Baldwin S, Chan G*, et al*: Antibody-mediated FOXP3 protein therapy induces apoptosis in cancer cells in vitro and inhibits metastasis in vivo. International journal of oncology 35: 167-173, 2009.

8. Ishigami S, Arigami T, Uenosono Y*, et al*: Cancerous HLA class I expression and regulatory T cell infiltration in gastric cancer. Cancer immunology, immunotherapy : CII 61: 1663-1669, 2012.

9. Jin JO, Zhang W, Wong KW, Kwak M, van Driel IR and Yu Q: Inhibition of breast cancer resistance protein (ABCG2) in human myeloid dendritic cells induces potent tolerogenic functions during LPS stimulation. PloS one 9: e104753, 2014.

10. Karanikas V, Speletas M, Zamanakou M*, et al*: Foxp3 expression in human cancer cells. Journal of translational medicine 6: 19, 2008.

11. Karkada M, Weir GM, Quinton T*, et al*: A novel breast/ovarian cancer peptide vaccine platform that promotes specific type-1 but not Treg/Tr1-type responses. Journal of immunotherapy (Hagerstown, Md : 1997) 33: 250-261, 2010.

12. Katoh H, Qin ZS, Liu R*, et al*: FOXP3 orchestrates H4K16 acetylation and H3K4 trimethylation for activation of multiple genes by recruiting MOF and causing displacement of PLU-1. Molecular cell 44: 770-784, 2011.

13. Lindenberg JJ, Oosterhoff D, Sombroek CC*, et al*: IL-10 conditioning of human skin affects the distribution of migratory dendritic cell subsets and functional T cell differentiation. PloS one 8: e70237, 2013.

14. Liyanage UK, Goedegebuure PS, Moore TT*, et al*: Increased prevalence of regulatory T cells (Treg) is induced by pancreas adenocarcinoma. Journal of immunotherapy (Hagerstown, Md : 1997) 29: 416-424, 2006.

15. Lu H, Wagner WM, Gad E*, et al*: Treatment failure of a TLR-7 agonist occurs due to self-regulation of acute inflammation and can be overcome by IL-10 blockade. Journal of immunology (Baltimore, Md : 1950) 184: 5360-5367, 2010.

16. Nakashima H, Fujisawa T, Husain SR and Puri RK: Interleukin-13 receptor alpha2 DNA prime boost vaccine induces tumor immunity in murine tumor models. Journal of translational medicine 8: 116, 2010.

17. Noori S, Hassan ZM, Mohammadi M, Habibi Z, Sohrabi N and Bayanolhagh S: Sclareol modulates the Treg intra-tumoral infiltrated cell and inhibits tumor growth in vivo. Cellular immunology 263: 148-153, 2010.

18. Qin A, Wen Z, Zhou Y*, et al*: MicroRNA-126 regulates the induction and function of CD4(+) Foxp3(+) regulatory T cells through PI3K/AKT pathway. Journal of cellular and molecular medicine 17: 252-264, 2013.

19. Rech AJ, Mick R, Martin S*, et al*: CD25 blockade depletes and selectively reprograms regulatory T cells in concert with immunotherapy in cancer patients. Science translational medicine 4: 134ra162, 2012.

20. Sugihara AQ, Rolle CE and Lesniak MS: Regulatory T cells actively infiltrate metastatic brain tumors. International journal of oncology 34: 1533-1540, 2009.

21. Tsai BY, Suen JL and Chiang BL: Lentiviral-mediated Foxp3 RNAi suppresses tumor growth of regulatory T cell-like leukemia in a murine tumor model. Gene therapy 17: 972-979, 2010.

22. Weiss VL, Lee TH, Song H*, et al*: Trafficking of high avidity HER-2/neu-specific T cells into HER-2/neu-expressing tumors after depletion of effector/memory-like regulatory T cells. PloS one 7: e31962, 2012.

23. Zhang HY and Sun H: Up-regulation of Foxp3 inhibits cell proliferation, migration and invasion in epithelial ovarian cancer. Cancer letters 287: 91-97, 2010.

24. Zhang M, Berndt BE, Chen JJ and Kao JY: Expression of a soluble TGF-beta receptor by tumor cells enhances dendritic cell/tumor fusion vaccine efficacy. Journal of immunology (Baltimore, Md : 1950) 181: 3690-3697, 2008.

25. Zhou J, Bashey A, Zhong R*, et al*: CTLA-4 blockade following relapse of malignancy after allogeneic stem cell transplantation is associated with T cell activation but not with increased levels of T regulatory cells. Biology of blood and marrow transplantation : journal of the American Society for Blood and Marrow Transplantation 17: 682-692, 2011.

26. Zlobec I, Minoo P, Terracciano L, Baker K and Lugli A: Characterization of the immunological microenvironment of tumour buds and its impact on prognosis in mismatch repair-proficient and -deficient colorectal cancers. Histopathology 59: 482-495, 2011.

**Laboratory research articles**

1. Knutson KL, Dang Y, Lu H*, et al*: IL-2 immunotoxin therapy modulates tumor-associated regulatory T cells and leads to lasting immune-mediated rejection of breast cancers in neu-transgenic mice. Journal of immunology (Baltimore, Md : 1950) 177: 84-91, 2006.

2. Matsuura K, Yamaguchi Y, Ueno H, Osaki A, Arihiro K and Toge T: Maturation of dendritic cells and T-cell responses in sentinel lymph nodes from patients with breast carcinoma. Cancer 106: 1227-1236, 2006.

3. Leong PP, Mohammad R, Ibrahim N*, et al*: Phenotyping of lymphocytes expressing regulatory and effector markers in infiltrating ductal carcinoma of the breast. Immunology letters 102: 229-236, 2006.

4. Zuo T, Liu R, Zhang H*, et al*: FOXP3 is a novel transcriptional repressor for the breast cancer oncogene SKP2. The Journal of clinical investigation 117: 3765-3773, 2007.

5. Hegyesi H, Colombo L, Pallinger E*, et al*: Impact of systemic histamine deficiency on the crosstalk between mammary adenocarcinoma and T cells. Journal of pharmacological sciences 105: 66-73, 2007.

6. Zuo T, Wang L, Morrison C*, et al*: FOXP3 is an X-linked breast cancer suppressor gene and an important repressor of the HER-2/ErbB2 oncogene. Cell 129: 1275-1286, 2007.

7. Vasir B, Wu Z, Crawford K*, et al*: Fusions of dendritic cells with breast carcinoma stimulate the expansion of regulatory T cells while concomitant exposure to IL-12, CpG oligodeoxynucleotides, and anti-CD3/CD28 promotes the expansion of activated tumor reactive cells. Journal of immunology (Baltimore, Md : 1950) 181: 808-821, 2008.

8. Lichtor T, Glick RP, Feldman LA*, et al*: Enhanced immunity to intracerebral breast cancer in mice immunized with a cDNA-based vaccine enriched for immunotherapeutic cells. Journal of immunotherapy (Hagerstown, Md : 1997) 31: 18-27, 2008.

9. Matsuura K, Yamaguchi Y, Osaki A*, et al*: FOXP3 expression of micrometastasis-positive sentinel nodes in breast cancer patients. Oncology reports 22: 1181-1187, 2009.

10. Liu Y, Wang Y, Li W, Zheng P and Liu Y: Activating transcription factor 2 and c-Jun-mediated induction of FoxP3 for experimental therapy of mammary tumor in the mouse. Cancer research 69: 5954-5960, 2009.

11. Horlock C, Stott B, Dyson PJ*, et al*: The effects of trastuzumab on the CD4+CD25+FoxP3+ and CD4+IL17A+ T-cell axis in patients with breast cancer. British journal of cancer 100: 1061-1067, 2009.

12. Liu R, Wang L, Chen G*, et al*: FOXP3 up-regulates p21 expression by site-specific inhibition of histone deacetylase 2/histone deacetylase 4 association to the locus. Cancer research 69: 2252-2259, 2009.

13. Raskin L, Rennert G and Gruber SB: FOXP3 germline polymorphisms are not associated with risk of breast cancer. Cancer genetics and cytogenetics 190: 40-42, 2009.

14. Generali D, Bates G, Berruti A*, et al*: Immunomodulation of FOXP3+ regulatory T cells by the aromatase inhibitor letrozole in breast cancer patients. Clinical cancer research : an official journal of the American Association for Cancer Research 15: 1046-1051, 2009.

15. Fahmi T, Esendagli G, Yilmaz G, Kansu E and Guc D: Immune compartmentalization of T cell subsets in chemically-induced breast cancer. Scandinavian journal of immunology 72: 339-348, 2010.

16. Gates JD, Clifton GT, Benavides LC*, et al*: Circulating regulatory T cells (CD4+CD25+FOXP3+) decrease in breast cancer patients after vaccination with a modified MHC class II HER2/neu (AE37) peptide. Vaccine 28: 7476-7482, 2010.

17. Xu L, Zhou Y, Xiao DM, Qin M, Luo JM and Tang XY: [The change of CD4+ CD25high CCR6+ regulatory T cells in breast cancer patients]. Sichuan da xue xue bao Yi xue ban = Journal of Sichuan University Medical science edition 41: 415-419, 2010.

18. Chen HX, Lin A, Shen CJ*, et al*: Upregulation of human leukocyte antigen-G expression and its clinical significance in ductal breast cancer. Human immunology 71: 892-898, 2010.

19. Pakravan N, Langroudi L, Hajimoradi M and Hassan ZM: Co-administration of GP96 and Her2/neu DNA vaccine in a Her2 breast cancer model. Cell stress & chaperones 15: 977-984, 2010.

20. Jaberipour M, Habibagahi M, Hosseini A, Habibabad SR, Talei A and Ghaderi A: Increased CTLA-4 and FOXP3 transcripts in peripheral blood mononuclear cells of patients with breast cancer. Pathology oncology research : POR 16: 547-551, 2010.

21. de Kruijf EM, van Nes JG, Sajet A*, et al*: The predictive value of HLA class I tumor cell expression and presence of intratumoral Tregs for chemotherapy in patients with early breast cancer. Clinical cancer research : an official journal of the American Association for Cancer Research 16: 1272-1280, 2010.

22. Joffroy CM, Buck MB, Stope MB, Popp SL, Pfizenmaier K and Knabbe C: Antiestrogens induce transforming growth factor beta-mediated immunosuppression in breast cancer. Cancer research 70: 1314-1322, 2010.

23. Wiedermann U, Wiltschke C, Jasinska J*, et al*: A virosomal formulated Her-2/neu multi-peptide vaccine induces Her-2/neu-specific immune responses in patients with metastatic breast cancer: a phase I study. Breast cancer research and treatment 119: 673-683, 2010.

24. Jung DJ, Jin DH, Hong SW*, et al*: Foxp3 expression in p53-dependent DNA damage responses. The Journal of biological chemistry 285: 7995-8002, 2010.

25. Rech AJ, Mick R, Kaplan DE, Chang KM, Domchek SM and Vonderheide RH: Homeostasis of peripheral FoxP3(+) CD4 (+) regulatory T cells in patients with early and late stage breast cancer. Cancer immunology, immunotherapy : CII 59: 599-607, 2010.

26. Yu J, Sun J, Wang SE*, et al*: Upregulated expression of indoleamine 2, 3-dioxygenase in primary breast cancer correlates with increase of infiltrated regulatory T cells in situ and lymph node metastasis. Clinical & developmental immunology 2011: 469135, 2011.

27. Anz D, Eiber S, Scholz C*, et al*: In breast cancer, a high ratio of tumour-infiltrating intraepithelial CD8+ to FoxP3+ cells is characteristic for the medullary subtype. Histopathology 59: 965-974, 2011.

28. Li CH, Kuo WH, Chang WC, Huang SC, Chang KJ and Sheu BC: Activation of regulatory T cells instigates functional down-regulation of cytotoxic T lymphocytes in human breast cancer. Immunologic research 51: 71-79, 2011.

29. Sun J, Yu J, Li H*, et al*: Upregulated expression of indoleamine 2, 3-dioxygenase in CHO cells induces apoptosis of competent T cells and increases proportion of Treg cells. Journal of experimental & clinical cancer research : CR 30: 82, 2011.

30. Farsam V, Hassan ZM, Zavaran-Hosseini A, Noori S, Mahdavi M and Ranjbar M: Antitumor and immunomodulatory properties of artemether and its ability to reduce CD4+ CD25+ FoxP3+ T reg cells in vivo. International immunopharmacology 11: 1802-1808, 2011.

31. Noori S and Hassan ZM: Dihydroartemisinin shift the immune response towards Th1, inhibit the tumor growth in vitro and in vivo. Cellular immunology 271: 67-72, 2011.

32. West NR, Panet-Raymond V, Truong PT*, et al*: Intratumoral Immune Responses Can Distinguish New Primary and True Recurrence Types of Ipsilateral Breast Tumor Recurrences (IBTR). Breast cancer : basic and clinical research 5: 105-115, 2011.

33. Olkhanud PB, Damdinsuren B, Bodogai M*, et al*: Tumor-evoked regulatory B cells promote breast cancer metastasis by converting resting CD4(+) T cells to T-regulatory cells. Cancer research 71: 3505-3515, 2011.

34. Haraldsdottir KH, Ivarsson K, Jansner K, Stenram U and Tranberg KG: Changes in immunocompetent cells after interstitial laser thermotherapy of breast cancer. Cancer immunology, immunotherapy : CII 60: 847-856, 2011.

35. Xiong Z and Ohlfest JR: Topical imiquimod has therapeutic and immunomodulatory effects against intracranial tumors. Journal of immunotherapy (Hagerstown, Md : 1997) 34: 264-269, 2011.

36. Tan W, Zhang W, Strasner A*, et al*: Tumour-infiltrating regulatory T cells stimulate mammary cancer metastasis through RANKL-RANK signalling. Nature 470: 548-553, 2011.

37. Li W, Wang L, Katoh H, Liu R, Zheng P and Liu Y: Identification of a tumor suppressor relay between the FOXP3 and the Hippo pathways in breast and prostate cancers. Cancer research 71: 2162-2171, 2011.

38. Tadmor T, Zhang Y, Cho HM, Podack ER and Rosenblatt JD: The absence of B lymphocytes reduces the number and function of T-regulatory cells and enhances the anti-tumor response in a murine tumor model. Cancer immunology, immunotherapy : CII 60: 609-619, 2011.

39. Razmkhah M, Jaberipour M, Erfani N, Habibagahi M, Talei AR and Ghaderi A: Adipose derived stem cells (ASCs) isolated from breast cancer tissue express IL-4, IL-10 and TGF-beta1 and upregulate expression of regulatory molecules on T cells: do they protect breast cancer cells from the immune response? Cellular immunology 266: 116-122, 2011.

40. Geller MA, Cooley S, Judson PL*, et al*: A phase II study of allogeneic natural killer cell therapy to treat patients with recurrent ovarian and breast cancer. Cytotherapy 13: 98-107, 2011.

41. Chan MS, Wang L, Felizola SJ*, et al*: Changes of tumor infiltrating lymphocyte subtypes before and after neoadjuvant endocrine therapy in estrogen receptor-positive breast cancer patients--an immunohistochemical study of Cd8+ and Foxp3+ using double immunostaining with correlation to the pathobiological response of the patients. The International journal of biological markers 27: e295-304, 2012.

42. Ma C, Zhang Q, Ye J*, et al*: Tumor-infiltrating gammadelta T lymphocytes predict clinical outcome in human breast cancer. Journal of immunology (Baltimore, Md : 1950) 189: 5029-5036, 2012.

43. Choi YJ, Yang KM, Kim SD*, et al*: Resveratrol analogue HS-1793 induces the modulation of tumor-derived T cells. Experimental and therapeutic medicine 3: 592-598, 2012.

44. Sisirak V, Faget J, Gobert M*, et al*: Impaired IFN-alpha production by plasmacytoid dendritic cells favors regulatory T-cell expansion that may contribute to breast cancer progression. Cancer research 72: 5188-5197, 2012.

45. Schmidt MA, Fortsch C, Schmidt M, Rau TT, Fietkau R and Distel LV: Circulating regulatory T cells of cancer patients receiving radiochemotherapy may be useful to individualize cancer treatment. Radiotherapy and oncology : journal of the European Society for Therapeutic Radiology and Oncology 104: 131-138, 2012.

46. Sekar D, Hahn C, Brune B, Roberts E and Weigert A: Apoptotic tumor cells induce IL-27 release from human DCs to activate Treg cells that express CD69 and attenuate cytotoxicity. European journal of immunology 42: 1585-1598, 2012.

47. Ramos RN, Chin LS, Dos Santos AP, Bergami-Santos PC, Laginha F and Barbuto JA: Monocyte-derived dendritic cells from breast cancer patients are biased to induce CD4+CD25+Foxp3+ regulatory T cells. Journal of leukocyte biology 92: 673-682, 2012.

48. Krausz LT, Fischer-Fodor E, Major ZZ and Fetica B: GITR-expressing regulatory T-cell subsets are increased in tumor-positive lymph nodes from advanced breast cancer patients as compared to tumor-negative lymph nodes. International journal of immunopathology and pharmacology 25: 59-66, 2012.

49. Lofdahl B, Ahlin C, Holmqvist M*, et al*: Inflammatory cells in node-negative breast cancer. Acta oncologica (Stockholm, Sweden) 51: 680-686, 2012.

50. van Pul KM, Vuylsteke RJ, Bril H, Stockmann HB and de Gruijl TD: Feasibility of flowcytometric quantitation of immune effector cell subsets in the sentinel lymph node of the breast after cryopreservation. Journal of immunological methods 375: 189-195, 2012.

51. Laumbacher B, Gu S and Wank R: Activated monocytes prime naive T cells against autologous cancer: vigorous cancer destruction in vitro and in vivo. Scandinavian journal of immunology 75: 314-328, 2012.

52. McInnes N, Sadlon TJ, Brown CY*, et al*: FOXP3 and FOXP3-regulated microRNAs suppress SATB1 in breast cancer cells. Oncogene 31: 1045-1054, 2012.

53. Petricevic B, Laengle J, Singer J*, et al*: Trastuzumab mediates antibody-dependent cell-mediated cytotoxicity and phagocytosis to the same extent in both adjuvant and metastatic HER2/neu breast cancer patients. Journal of translational medicine 11: 307, 2013.

54. Hossain DM, Panda AK, Manna A*, et al*: FoxP3 acts as a cotranscription factor with STAT3 in tumor-induced regulatory T cells. Immunity 39: 1057-1069, 2013.

55. Noori S, Hassan ZM, Yaghmaei B and Dolatkhah M: Antitumor and immunomodulatory effects of salvigenin on tumor bearing mice. Cellular immunology 286: 16-21, 2013.

56. Bos PD, Plitas G, Rudra D, Lee SY and Rudensky AY: Transient regulatory T cell ablation deters oncogene-driven breast cancer and enhances radiotherapy. The Journal of experimental medicine 210: 2435-2466, 2013.

57. Lee-Chang C, Bodogai M, Martin-Montalvo A*, et al*: Inhibition of breast cancer metastasis by resveratrol-mediated inactivation of tumor-evoked regulatory B cells. Journal of immunology (Baltimore, Md : 1950) 191: 4141-4151, 2013.

58. Allard B, Pommey S, Smyth MJ and Stagg J: Targeting CD73 enhances the antitumor activity of anti-PD-1 and anti-CTLA-4 mAbs. Clinical cancer research : an official journal of the American Association for Cancer Research 19: 5626-5635, 2013.

59. Jeong MH, Lee CM, Lee SW*, et al*: Cordycepin-enriched Cordyceps militaris induces immunomodulation and tumor growth delay in mouse-derived breast cancer. Oncology reports 30: 1996-2002, 2013.

60. Ljujic B, Milovanovic M, Volarevic V*, et al*: Human mesenchymal stem cells creating an immunosuppressive environment and promote breast cancer in mice. Scientific reports 3: 2298, 2013.

61. Salatino M, Dalotto-Moreno T and Rabinovich GA: Thwarting galectin-induced immunosuppression in breast cancer. Oncoimmunology 2: e24077, 2013.

62. He YJ, Zhou J, Zhao TF*, et al*: Eps8 vaccine exerts prophylactic antitumor effects in a murine model: a novel vaccine for breast carcinoma. Molecular medicine reports 8: 662-668, 2013.

63. Wang HY, Shi QF, Sun Y, He JJ and Wang YL: [Tumor infiltrating regulatory T cells in human breast cancer and associated draining lymph nodes: an in-situ analysis]. Zhonghua bing li xue za zhi Chinese journal of pathology 42: 95-100, 2013.

64. Shen Z, Chen L, Yang X*, et al*: Downregulation of Ezh2 methyltransferase by FOXP3: new insight of FOXP3 into chromatin remodeling? Biochimica et biophysica acta 1833: 2190-2200, 2013.

65. Biragyn A, Bodogai M, Olkhanud PB*, et al*: Inhibition of lung metastasis by chemokine CCL17-mediated in vivo silencing of genes in CCR4+ Tregs. Journal of immunotherapy (Hagerstown, Md : 1997) 36: 258-267, 2013.

66. Benevides L, Cardoso CR, Tiezzi DG, Marana HR, Andrade JM and Silva JS: Enrichment of regulatory T cells in invasive breast tumor correlates with the upregulation of IL-17A expression and invasiveness of the tumor. European journal of immunology 43: 1518-1528, 2013.

67. Hamidinia M, Ghafourian Boroujerdnia M, Talaiezadeh A, Solgi G, Taghdiri M and Khodadadi A: Concomitant Increase of OX40 and FOXP3 transcripts in peripheral blood of patients with breast cancer. Iranian journal of immunology : IJI 10: 22-30, 2013.

68. Noori S, Hassan ZM and Salehian O: Sclareol reduces CD4+ CD25+ FoxP3+ Treg cells in a breast cancer model in vivo. Iranian journal of immunology : IJI 10: 10-21, 2013.

69. Kawabata A, Ohta N, Seiler G*, et al*: Naive rat umbilical cord matrix stem cells significantly attenuate mammary tumor growth through modulation of endogenous immune responses. Cytotherapy 15: 586-597, 2013.

70. Yu J, Du W, Yan F*, et al*: Myeloid-derived suppressor cells suppress antitumor immune responses through IDO expression and correlate with lymph node metastasis in patients with breast cancer. Journal of immunology (Baltimore, Md : 1950) 190: 3783-3797, 2013.

71. Zheng J, Deng J, Jiang L*, et al*: Heterozygous genetic variations of FOXP3 in Xp11.23 elevate breast cancer risk in Chinese population via skewed X-chromosome inactivation. Human mutation 34: 619-628, 2013.

72. Soliman H, Rawal B, Fulp J*, et al*: Analysis of indoleamine 2-3 dioxygenase (IDO1) expression in breast cancer tissue by immunohistochemistry. Cancer immunology, immunotherapy : CII 62: 829-837, 2013.

73. Nair S, Aldrich AJ, McDonnell E*, et al*: Immunologic targeting of FOXP3 in inflammatory breast cancer cells. PloS one 8: e53150, 2013.

74. Fu Z, Zuo Y, Li D*, et al*: The crosstalk: Tumor-infiltrating lymphocytes rich in regulatory T cells suppressed cancer-associated fibroblasts. Acta oncologica (Stockholm, Sweden) 52: 1760-1770, 2013.

75. Verma C, Eremin JM, Robins A*, et al*: Abnormal T regulatory cells (Tregs: FOXP3+, CTLA-4+), myeloid-derived suppressor cells (MDSCs: monocytic, granulocytic) and polarised T helper cell profiles (Th1, Th2, Th17) in women with large and locally advanced breast cancers undergoing neoadjuvant chemotherapy (NAC) and surgery: failure of abolition of abnormal treg profile with treatment and correlation of treg levels with pathological response to NAC. Journal of translational medicine 11: 16, 2013.

76. Li W, Katoh H, Wang L*, et al*: FOXP3 regulates sensitivity of cancer cells to irradiation by transcriptional repression of BRCA1. Cancer research 73: 2170-2180, 2013.

77. Mulligan AM, Raitman I, Feeley L*, et al*: Tumoral lymphocytic infiltration and expression of the chemokine CXCL10 in breast cancers from the Ontario Familial Breast Cancer Registry. Clinical cancer research : an official journal of the American Association for Cancer Research 19: 336-346, 2013.

78. Dalotto-Moreno T, Croci DO, Cerliani JP*, et al*: Targeting galectin-1 overcomes breast cancer-associated immunosuppression and prevents metastatic disease. Cancer research 73: 1107-1117, 2013.

79. Zheng X, Koropatnick J, Chen D*, et al*: Silencing IDO in dendritic cells: a novel approach to enhance cancer immunotherapy in a murine breast cancer model. International journal of cancer Journal international du cancer 132: 967-977, 2013.

80. Tiriveedhi V, Fleming TP, Goedegebuure PS*, et al*: Mammaglobin-A cDNA vaccination of breast cancer patients induces antigen-specific cytotoxic CD4+ICOShi T cells. Breast cancer research and treatment 138: 109-118, 2013.

81. Kim ST, Jeong H, Woo OH*, et al*: Tumor-infiltrating lymphocytes, tumor characteristics, and recurrence in patients with early breast cancer. American journal of clinical oncology 36: 224-231, 2013.

82. Gong L, Qin Q, Zhou L*, et al*: Effects of fentanyl anesthesia and sufentanil anesthesia on regulatory T cells frequencies. International journal of clinical and experimental pathology 7: 7708-7716, 2014.

83. Douglass S, Meeson AP, Overbeck-Zubrzycka D*, et al*: Breast cancer metastasis: demonstration that FOXP3 regulates CXCR4 expression and the response to CXCL12. The Journal of pathology 234: 74-85, 2014.

84. Chan MS, Chen SF, Felizola SJ*, et al*: Correlation of tumor-infiltrative lymphocyte subtypes alteration with neoangiogenesis before and after neoadjuvant chemotherapy treatment in breast cancer patients. The International journal of biological markers 29: e193-203, 2014.

85. Tymoszuk P, Charoentong P, Hackl H*, et al*: High STAT1 mRNA levels but not its tyrosine phosphorylation are associated with macrophage infiltration and bad prognosis in breast cancer. BMC cancer 14: 257, 2014.

86. Chen X, Yang Y, Zhou Q*, et al*: Effective chemoimmunotherapy with anti-TGFbeta antibody and cyclophosphamide in a mouse model of breast cancer. PloS one 9: e85398, 2014.

87. Liu T, Song YN, Shi QY, Liu Y, Bai XN and Pang D: Study of circulating antibodies against CD25 and FOXP3 in breast cancer. Tumour biology : the journal of the International Society for Oncodevelopmental Biology and Medicine 35: 3779-3783, 2014.

88. Jahan P, Ramachander VR, Maruthi G, Nalini S, Latha KP and Murthy TS: Foxp3 promoter polymorphism (rs3761548) in breast cancer progression: a study from India. Tumour biology : the journal of the International Society for Oncodevelopmental Biology and Medicine 35: 3785-3791, 2014.

89. Jovanovic IP, Pejnovic NN, Radosavljevic GD*, et al*: Interleukin-33/ST2 axis promotes breast cancer growth and metastases by facilitating intratumoral accumulation of immunosuppressive and innate lymphoid cells. International journal of cancer Journal international du cancer 134: 1669-1682, 2014.

90. Duechler M, Peczek L, Zuk K, Zalesna I, Jeziorski A and Czyz M: The heterogeneous immune microenvironment in breast cancer is affected by hypoxia-related genes. Immunobiology 219: 158-165, 2014.

91. Martinson HA, Jindal S, Durand-Rougely C, Borges VF and Schedin P: Wound healing-like immune program facilitates postpartum mammary gland involution and tumor progression. International journal of cancer Journal international du cancer 136: 1803-1813, 2015.

**Lack sufficient data**

1. Bohling SD and Allison KH: Immunosuppressive regulatory T cells are associated with aggressive breast cancer phenotypes: a potential therapeutic target. Modern pathology : an official journal of the United States and Canadian Academy of Pathology, Inc 21: 1527-1532, 2008.

2. Ohara M, Yamaguchi Y, Matsuura K, Murakami S, Arihiro K and Okada M: Possible involvement of regulatory T cells in tumor onset and progression in primary breast cancer. Cancer immunology, immunotherapy : CII 58: 441-447, 2009.

3. Tsang JY, Hui SW, Ni YB, et al: Lymphocytic infiltrate is associated with favorable biomarkers profile in HER2-overexpressing breast cancers and adverse biomarker profile in ER-positive breast cancers. Breast cancer research and treatment 143: 1-9, 2014.

**Not relevant to the prognostic value of FOXP3**

1. Abo-Elenein A, Elgohary SE, Hashish A and El-Halaby E: Significance of immunoregulatory T cells in different stages of breast cancer patients. The Egyptian journal of immunology / Egyptian Association of Immunologists 15: 145-152, 2008.

2. Cimino-Mathews A, Ye X, Meeker A, Argani P and Emens LA: Metastatic triple-negative breast cancers at first relapse have fewer tumor-infiltrating lymphocytes than their matched primary breast tumors: a pilot study. Human pathology 44: 2055-2063, 2013.

3. Droeser RA, Obermann EC, Wolf AM, Wallner S, Wolf D and Tzankov A: Negligible nuclear FOXP3 expression in breast cancer epithelial cells compared with FOXP3-positive T cells. Clinical breast cancer 13: 264-270, 2013.

4. Gokmen-Polar Y, Thorat MA, Sojitra P, Saxena R and Badve S: FOXP3 expression and nodal metastasis of breast cancer. Cellular oncology (Dordrecht) 36: 405-409, 2013.

5. Gupta R, Babb JS, Singh B*, et al*: The numbers of FoxP3+ lymphocytes in sentinel lymph nodes of breast cancer patients correlate with primary tumor size but not nodal status. Cancer investigation 29: 419-425, 2011.

6. Gupta S, Joshi K, Wig JD and Arora SK: Intratumoral FOXP3 expression in infiltrating breast carcinoma: Its association with clinicopathologic parameters and angiogenesis. Acta oncologica (Stockholm, Sweden) 46: 792-797, 2007.

7. Ladoire S, Arnould L, Apetoh L*, et al*: Pathologic complete response to neoadjuvant chemotherapy of breast carcinoma is associated with the disappearance of tumor-infiltrating foxp3+ regulatory T cells. Clinical cancer research : an official journal of the American Association for Cancer Research 14: 2413-2420, 2008.

8. Lal A, Chan L, Devries S*, et al*: FOXP3-positive regulatory T lymphocytes and epithelial FOXP3 expression in synchronous normal, ductal carcinoma in situ, and invasive cancer of the breast. Breast cancer research and treatment 139: 381-390, 2013.

9. Lee HJ, Seo JY, Ahn JH, Ahn SH and Gong G: Tumor-associated lymphocytes predict response to neoadjuvant chemotherapy in breast cancer patients. Journal of breast cancer 16: 32-39, 2013.

10. Li Y, Gao P, Yang J, Yu H, Zhu Y and Si W: Relationship between IL-10 expression and prognosis in patients with primary breast cancer. Tumour biology : the journal of the International Society for Oncodevelopmental Biology and Medicine 35: 11533-11540, 2014.

11. Li YQ, Liu FF, Zhang XM, Guo XJ, Ren MJ and Fu L: Tumor secretion of CCL22 activates intratumoral Treg infiltration and is independent prognostic predictor of breast cancer. PloS one 8: e76379, 2013.

12. Lopes LF, Guembarovski RL, Guembarovski AL*, et al*: FOXP3 transcription factor: a candidate marker for susceptibility and prognosis in triple negative breast cancer. BioMed research international 2014: 341654, 2014.

13. Mansfield AS, Heikkila PS, Vaara AT, von Smitten KA, Vakkila JM and Leidenius MH: Simultaneous Foxp3 and IDO expression is associated with sentinel lymph node metastases in breast cancer. BMC cancer 9: 231, 2009.

14. Nakamura R, Sakakibara M, Nagashima T*, et al*: Accumulation of regulatory T cells in sentinel lymph nodes is a prognostic predictor in patients with node-negative breast cancer. European journal of cancer (Oxford, England : 1990) 45: 2123-2131, 2009.

15. Seo AN, Lee HJ, Kim EJ*, et al*: Tumour-infiltrating CD8+ lymphocytes as an independent predictive factor for pathological complete response to primary systemic therapy in breast cancer. British journal of cancer 109: 2705-2713, 2013.

16. Sisirak V, Faget J, Vey N*, et al*: Plasmacytoid dendritic cells deficient in IFNalpha production promote the amplification of FOXP3 regulatory T cells and are associated with poor prognosis in breast cancer patients. Oncoimmunology 2: e22338, 2013.

17. Xu L, Xu W, Qiu S and Xiong S: Enrichment of CCR6+Foxp3+ regulatory T cells in the tumor mass correlates with impaired CD8+ T cell function and poor prognosis of breast cancer. Clinical immunology (Orlando, Fla) 135: 466-475, 2010.

**Not operable breast cancer**

1. Aruga T, Suzuki E, Saji S*, et al*: A low number of tumor-infiltrating FOXP3-positive cells during primary systemic chemotherapy correlates with favorable anti-tumor response in patients with breast cancer. Oncology reports 22: 273-278, 2009.

2. Demir L, Yigit S, Ellidokuz H*, et al*: Predictive and prognostic factors in locally advanced breast cancer: effect of intratumoral FOXP3+ Tregs. Clinical & experimental metastasis 30: 1047-1062, 2013.

3. Ladoire S, Arnould L, Mignot G*, et al*: Presence of Foxp3 expression in tumor cells predicts better survival in HER2-overexpressing breast cancer patients treated with neoadjuvant chemotherapy. Breast cancer research and treatment 125: 65-72, 2011.

4. Ladoire S, Mignot G, Dabakuyo S*, et al*: In situ immune response after neoadjuvant chemotherapy for breast cancer predicts survival. The Journal of pathology 224: 389-400, 2011.

5. Lee S, Cho EY, Park YH, Ahn JS and Im YH: Prognostic impact of FOXP3 expression in triple-negative breast cancer. Acta oncologica (Stockholm, Sweden) 52: 73-81, 2013.

6. Liu F, Li Y, Ren M*, et al*: Peritumoral FOXP3(+) regulatory T cell is sensitive to chemotherapy while intratumoral FOXP3(+) regulatory T cell is prognostic predictor of breast cancer patients. Breast cancer research and treatment 135: 459-467, 2012.

7. Oda N, Shimazu K, Naoi Y*, et al*: Intratumoral regulatory T cells as an independent predictive factor for pathological complete response to neoadjuvant paclitaxel followed by 5-FU/epirubicin/cyclophosphamide in breast cancer patients. Breast cancer research and treatment 136: 107-116, 2012.

**Duplicate study**

1. Mahmoud SM, Paish EC, Powe DG*, et al*: An evaluation of the clinical significance of FOXP3+ infiltrating cells in human breast cancer. Breast cancer research and treatment 127: 99-108, 2011.
