# Supplementary material for: The Prognostic Value of Forkhead Box P3 Expression in Operable Breast Cancer: A Large-Scale Meta-Analysis
Source: PLoS One. 2015 Aug 25;10(8):e0136374. doi: 10.1371/journal.pone.0136374 (PMC4549287; doi:10.1371/journal.pone.0136374)
Supplement: S1 Table — (Note: Data given as the definite option of each item. ※ means that the score was zero.) (DOCX) [file pone.0136374.s004.docx]

**S1 Table. The NOS scores of the included studies** (Note: Data given as the definite option of each item. ^※^ means that the score was zero.)

| **First author**  **[Ref no.]** | **(1)** | **Selection (2)** | **(3)** | **(4)** | **Comparability** | **(1)** | **Outcome**  **(2)** | **(3)** |
| --- | --- | --- | --- | --- | --- | --- | --- | --- |
| Liu et al. [6] | a | a | a | b | b | b | a | d |
| Ali et al. [7] | a | a | a | a | b | b | a | d |
| Kim et al. [8] | b | a | c | b | b | d | a | d |
| Takenaka et al. [9] | b | a | c | b | 0**^※^** | b | a | d |
| Maeda et al. [10] | b | a | a | b | b | c | a | d |
| Sun et al. [11] | b | a | c | a | b | b | a | d |
| Won et al. [12] | a | a | a | b | b | d | b | d |
| Kim et al. [13] | b | a | a | b | b | b | a | d |
| West et al. [14] | a | a | a | b | b | b | a | d |
| Bates et al. [15] | b | a | a | b | b | b | a | d |
| Droeser et al. [16] | b | a | c | b | b | b | a | d |
| Ladoire et al. [17] | a | a | a | b | b | b | a | d |
| Liu et al. [18] | b | a | b | b | 0**^※^** | b | a | d |
| Yan et al. [19] | b | a | b | b | 0**^※^** | b | a | d |
| Merlo et al. [20] | a | a | a | b | 0**^※^** | a | a | b |
| Gobert et al. [21] | b | a | c | b | b | b | a | d |

NOS: Newcastle–Ottawa Quality Assessment Scale.
